# Cranberry for Bacteriuria in Individuals with Spinal Cord Injury: A Systematic Review and Meta-Analysis

**DOI:** 10.1155/2020/9869851

**Published:** 2020-10-30

**Authors:** Anna Raguzzini, Elisabetta Toti, Tommaso Sciarra, Anna Lucia Fedullo, Ilaria Peluso

**Affiliations:** ^1^Research Centre for Food and Nutrition, Council for Agricultural Research and Economics (CREA-AN), Rome, Italy; ^2^Joint Veteran Center, Scientific Department, Army Medical Center, Rome, Italy

## Abstract

**Background:**

Urinary tract infection (UTI) is common in individuals with spinal cord injury (SCI) and neurogenic lower urinary tract dysfunction (NLUTD) and in veterans with SCI who use antibiotics improperly for asymptomatic bacteriuria. Cranberry (CB) has been suggested for UTI prevention.

**Methods:**

We performed a systematic search up to May 2020 in the following databases: AccessMedicine, BioMed Central, CINAHL, Cochrane Library, ProQuest, and PubMed. Quality assessment was performed using a specifically designed quality score. Risk ratio was calculated with both random effect model analysis (DerSimonian-Laird method) and quality effect model analysis (Doi Thalib method).

**Results:**

Six studies on bacteriuria and SCI were reviewed. From the four studies available for meta-analysis, two of which with individuals taking both CB and control, 477 data from 415 participants were analysed (241 CB and 236 control). No significant differences were detected with meta-analysis. However, bias, limitations, and incompleteness were observed in the reviewed studies.

**Conclusion:**

Although further studies are needed, we suggest an accurate monitoring of diet and fluid intake, the evaluation of risk for potential food or nutraceutical interactions with drugs, and the inclusion of inflammatory markers among the outcomes in addition to UTI.

## 1. Introduction

Spinal cord injury (SCI) is a damage to the spinal cord that may result in motor paralysis and sensory loss below the level of the lesion [1]. The highest documented global prevalence of SCI was found in the United States of America (906/million) and the lowest in France (250/million), while the incidence of traumatic SCI was highest in New Zealand (49.1/million) and lowest in Spain (8.0/million) [2]. In Italy, the incidence of traumatic SCI during 2013-2014 was 14.7/million per year, the mean age was 54 years old, and the male to female ratio was 4 : 1 [3]. High incidence was documented in veterans. In a systematic review of 25 articles, the overall incidence rate of war-related SCI varied from 4.3 to 5.6/10,000 person-years [4]. These veterans had predominantly thoracic or lumbar level, complete (American Spinal Injury Association [ASIA] Impairment Scale A) SCI, associated with other bodily injuries in 43.9-78.1% of cases [4]. Moreover, polypharmacy is common in veterans [5, 6], and nutraceutical-drug [5] and food-drug interactions [6] should be considered.

Neurogenic lower urinary tract dysfunction (NLUTD) can be observed in 83% of veterans with SCI and is associated with urinary tract infection (UTI) [7]. Results of routine testing reported that 69% of the urine cultures of veterans with SCI were positive for bacteria, but 87% were asymptomatic bacteriuria cases, of which 36% were treated with antibiotics [8]. Epidemiology of antibiotic resistance in veterans with SCI suggests improving prescribing of appropriate antibiotics [9]. There is a large consensus that antibiotic prophylaxis is not recommended for UTI [10–12] and that asymptomatic bacteriuria should not be treated with antibiotics [13].

Although cranberry (CB) may be effective in preventing UTI recurrence in women [14], data from meta-analyses have reported conflicting results, and it has been suggested that conclusions on cranberry and UTI should consider the differences among the populations studied [15]. Sappal et al. [16] recently investigated the effect of concentrated proanthocyanidins (PAC) from CB for reduction of bacteriuria in male veterans with SCI and did not find reduction of bacteriuria and pyuria or improvement in subjective urine quality.

### 1.1. Aim and Objectives

We aimed to evaluate the following hypothesis: CB products (including extracts) are more effective than placebo or no treatment in reducing bacteriuria and/or in the prevention of UTI in individuals with SCI. Furthermore, we aimed to suggest a specific quality assessment for studies with nutraceuticals involving individuals with SCI.

To this aim, we conducted a systematic review and evaluated previous meta-analysis risk of bias assessment [17–19]. Although it was not a meta-analysis, we also considered the systematic review of Navarrete-Opazo et al. [20], because it was focused on individuals with SCI.

## 2. Materials and Methods

### 2.1. Study Selection

**Figure 1:**
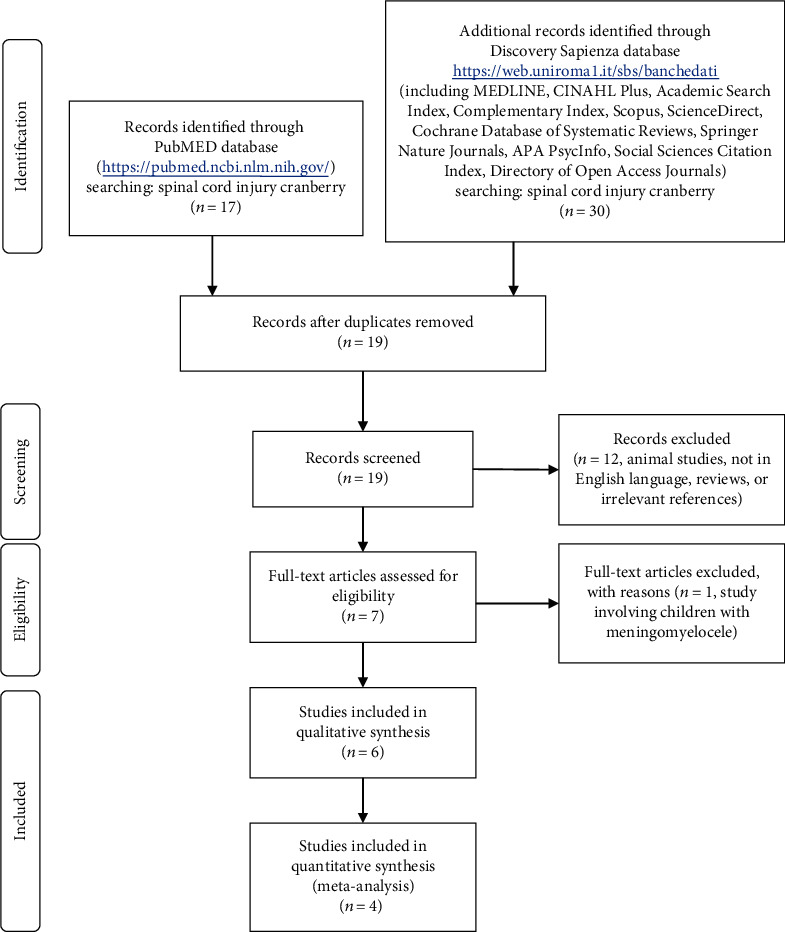
Four-phase flow diagram of systematic review and meta-analysis, according to the PRISMA Statement.

Figure 1 shows the four-phase diagram of meta-analysis (according to the PRISMA Statement) and the flow of the studies processed in this review. We performed a systematic search in PubMed and in the Discovery Sapienza/medicine pharmacy and psychology including (among others) the following databases: AccessMedicine, BioMed Central, CINAHL, Cochrane Library, and ProQuest, with the search terms spinal cord injury and cranberry, up to May 2020 (Figure 1).

Given that the aim of the present review was specifically SCI, interventions that involved individuals with other health conditions were excluded. In particular, we excluded studies involving subjects with spina bifida, who had higher urinary tumor growth factor *β*-1 than patients with SCI [21], and children with myelomeningocele [22], being children among the groups where CB products seemed to be more effective (relative risk: RR range -0.33 [18] -0.48 [17]). Moreover, these are two congenital conditions [23], and this review is aimed at evaluating the effect of CB in individuals with traumatic SCI.

All studies that met the following criteria were included in this review: studies that appear in an edited journal (peer-review criterion), published in English (language criterion), and focused on the effect of CB on bacteriuria or UTI versus control (topic criterion), regardless of the CB bioactive compounds' source and dose and the study design (parallel, crossover, controlled, and uncontrolled). First trials were identified through the title or abstract. Then, the full text of the article was obtained. Finally, based on inclusion and exclusion criteria, eligible studies were included (A.R. and E.T.).

### 2.2. Data Extraction and Quality Assessment

A data extraction form, including quality characteristics, was designed, and selected studies were reviewed by all authors. To ensure uniformity, data extraction was performed independently by two reviewers (A.R. and E.T.), and all data were entered by these reviewers. Discrepancies were resolved by discussion between the two reviewers, and unresolved disagreement was referred to a third reviewer (I.P.).

Table 1 shows previously reported judgments of studies (Table 1). The previously used (Table 1) assessment of bias tool includes random sequence generation (selection bias), allocation concealment (selection bias), blinding of participants and personnel (performance bias), blinding of outcome assessment (detection bias), incomplete outcome data (attrition bias), selective reporting (reporting bias), and other biases [17], each scored as “high risk,” “low risk,” or “unclear.” Navarrete-Opazo et al. [20] judged studies as unclear risk for reporting bias, because there was no previous registration of the protocols.

In this study, withdrawal was not considered as a bias, being the compliance of volunteers generally due to factors not imputable to researchers [24], according to the quality score previously used for intervention studies with flavonoids [24]. The score (range 0-1) [24] includes proper control (0.3), compliance assessment (0.1), dietary record (food records or food-frequency questionnaires throughout the study, 0.06), food antioxidant intake in subjects' selection criteria (flavonoid-rich food or antioxidant supplement consumption, 0.05), washout and/or run-in period (0.05, washout and/or run-in period for crossover studies only run-in for parallel design), marker of bioavailability (0.05), double blinding (0.05), no funding support (0.03, from profit companies), and no food/supplement donation (0.01).

Intra- and interstudy baseline comparability was removed from the previously suggested score [24] because no mean difference after versus before treatment was calculated, and the corresponding score 0.3 was divided into groups balanced for lesion level/urine collection (0.1), UTI diagnosis after treatment including autonomic dysreflexia (0.1), and comorbidity and drug use specified (0.1).

Discrepancies were resolved through discussions between the two authors who performed the quality assessment (A.R. and T.S.) or through consultation with a third investigator (I.P.).

### 2.3. Meta-Analysis

Two [16, 25] out of the 6 selected studies [16, 25–29] have been excluded from the meta-analysis since results were provided in the form of figures, but we retained them for discussion, as in the previous meta-analysis [24]. With the definition of UTI being different among the studies, we use bacteriuria as the outcome. Moreover, asymptomatic bacteriuria is often improperly treated with antibiotics [8].

Four studies [26–29] met the inclusion criteria and provided data for the analyses of CB versus control (Figure 1). Dichotomous outcomes (UTI cases in CB and control groups) from each study were collected in order to compute individual-study RR (with 95% confidence intervals). Random effect model analysis (DerSimonian-Laird method) and quality effect model analysis (Doi Thalib method, by using the quality score as the probability modifier) were reported. Number needed to treat (NNT) was estimated. Statistical heterogeneity was assessed by using the *Q* statistics for quality effect model and by *t*^2^ and prediction interval [30] for the random effect model.

In order to detect the presence of publication bias, funnel plot and Egger's weighted regression statistics were used. Symmetry/asymmetry of the funnel plot was defined through visual examination, and trim-and-fill sensitivity analysis was performed. Furthermore, we used the L'Abbe plot [31] in order to visualize the relationship between the effect of treatment and the bacteriuria.

## 3. Results

### 3.1. Description of Included Studies

Six studies were retrieved from the systematic search, of which 2 crossover, 3 parallel (two 2 arms and one 4 arms), and 1 longitudinal (water before washout and CB juice), with a total of 449 volunteers.

Characteristics of participants, including and excluding selection criteria, are shown in Table 2. Lesion level and ASIA classification were very variable among studies and, when reported, among groups within the same study (Table 2). Reported reasons for dropout/loss of follow-up included noncompliance with pill counts [29], developing of urinary stones [29], recurrent UTI [25], abdominal discomfort attributed to CB [25], and personal reasons, including travels [25].

Only 1 study evaluated the effect of CB juice, whereas the others evaluated CB tablets or capsules (Table 3). Treatment range between 1 week and 6 months and 2 out of 3 crossover studies included a washout period between the two periods of intervention (treatment and control), whereas the other studies did not include a run-in period. The 4-arm study also included a methenamine hippurate (MH) group and a CB+MH group. All except for 1 study did not report PAC content in the CB product. Dropout rates range from 0% to 43% and different dropout rates were reported for CB (15%), CB+MH (21%), placebo (13%), and MH (23%) in the 4-arm study.

The outcomes of studies were symptomatic UTI, bacteriuria plus pyuria (urinary white blood cells count: WBC), or bacteriuria only (Table 3). Among the studies, only Hess et al. [29] reported a significant effect on symptomatic UTI after consumption of CB tablet (1 g/d for 6 months) compared to placebo. On the other hand, Lee et al. [28] reported bowel dysfunctions (diarrhoea or constipation) in eleven participants, nausea in two, and rash in one, after the treatment with the CB capsule (2 g/d for 6 months).

### 3.2. Quality of the Included Studies

Disagreement, between the two authors who performed data extraction and quality assessment, occurred for the study of Lee et al. [28], who did not exclude from the follow-up patients who discontinued the intervention and coupled CB and CB+MH groups and placebo and MH groups for the analysis. After the consultation with the third reviewer, the study was included in the quantitative synthesis (Figure 1), due to the great number of subjects, but with the lower quality score, according with criteria in Table 4.

The most frequent limitations were no comorbidity and drug use specified (all studies), markers of bioavailability (all studies), and no compliance, diet, or antioxidant monitoring (Table 4).

Only 3 studies included autonomic dysfunction in the symptomatic UTI diagnostic criteria, and in 3 studies, the balancing between treatment and control for the lesion level and urinary collection (management of NLUTD) was not properly controlled.

In particular, Waites et al. [27] stated that groups were unbalanced for catheterization and Lee et al. [28] did not furnish information for the 4 arms of treatment, pooling CB and CB+MH in the treatment group and placebo and MH in the control group. This kind of analysis is a bias also for proper control (Table 4).

### 3.3. Meta-Analysis

**Figure 2:**
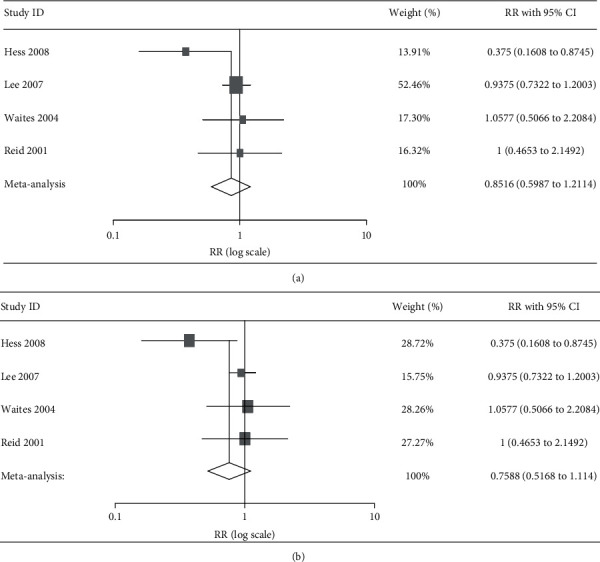
Meta-analysis: (a) forest plot of random effect model; (b) forest plot of quality effect model.

**Figure 3:**
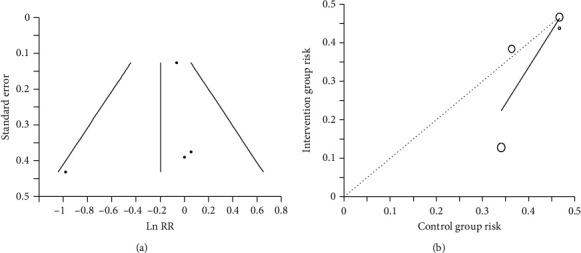
(a) Funnel plot. Ln RR: risk ratio logarithm. (b) L'Abbe plot. Dotted line: line of equality.

Since there were 2 studies with individuals taking both treatment and control, 1 crossover [29] and 1 longitudinal [26], 477 data from 415 participants were analysed in this meta-analysis. Overall, 241 subjects received CB and 236 control. No significant differences were detected with meta-analysis in random (*p* = 0.372) and quality (*p* = 0.415) effect models (Figure 2). NNT was 17 (95% CI 34.36 to 6.82), and low to high statistical heterogeneity was found for random and quality effect models, respectively (*t*^2^ 0.05, *Q* 75%, Egger intercept −0.880, *p* = 0.568). However, the 95% prediction interval ranged between 0.3 and 2.1. The funnel plot showed no asymmetric distribution of results (Figure 3(a)) and trim-and-fill analysis did not suggest potential publication bias. On the other hand, the visual inspection of the L'Abbe plot revealed that only half of the participants were at risk of UTI in the control group (Figure 3(b)).

## 4. Discussion

### 4.1. Summary of Main Results

Six studies were retrieved in the present systematic review, 4 of which reported data suitable for the meta-analysis (Figure 1). Considering that inclusion of low-quality studies may bias the estimated effect, while restriction to high-quality studies can reduce information, a quality score has been assigned and a quality effect model meta-analysis has been performed, in addition to the random effect model meta-analysis. We did not observe significant effect of CB products (Table 3) on bacteriuria in individuals with SCI (Table 2 and Figure 2).

About one in every 17 patients will benefit from the treatment. However, NNT does not account for a patient's baseline risk, probably different due to intra- and interstudy variability (Table 2). Moreover, the NNT of a given treatment will be very different when describing the value versus placebo instead of another active therapy (Tables 3 and 4; Lee et al. [28] did not furnish information for the 4 arms of treatment). Moreover, we observed a broad prediction interval (95% 0.3-2.1), suggesting a range of possible effects in relation to harm and clinical benefit thresholds (1, null effect) and indicating the existence of settings where the treatment has a suboptimal and possibly even harmful effect. None of the studies included in the quantitative synthesis involved the veterans who can be very different from the patients seen in all studies that have been done in the past. Therefore, the prediction interval cannot tell us what we might expect for these patients and specific studies are required to enable more informed clinical decision-making. Moreover, the prediction interval is affected by study bias. If bias exists, as summarized in Table 4, the effect sizes observed in future studies might occur beyond the limits of the prediction interval.

### 4.2. Completeness, Quality, and Applicability of Evidence

Navarrete-Opazo et al. [20], after a quality assessment across studies, carried out using the Grading of Recommendations Assessment, Development and Evaluation (GRADE), reported that overall, the studies were rated as moderate-quality evidence and the highest quality was found in the trial by Lee et al. [28]. From that, the authors concluded that the quality of the evidence was strong enough to reasonably conclude that cranberry supplementation is not effective for prevention of UTIs in people with SCI [20]. By using our risk of bias assessment (Table 4), we concluded that the quality of evidence is low due to the bias, limitations, and incompleteness of the reviewed studies.

Intersubjects' variability has been reported for absorption, metabolism, and excretion of CB polyphenols in healthy individuals, partly due to the variation in the gut microbiota [32]. Several studies have reported different intestinal microbiota in individuals with SCI compared to healthy controls [33], and diet is known to influence gut microbiota [33] and to affect UTI risk [34]. Therefore, monitoring food consumption is of great importance in those studies aimed at evaluating the effects of bioactive compounds from plant food. Mobile phone applications for dietary intake monitoring are available in many countries [35, 36], and it has been reported that a 12-hour dietary recall app was in good agreement with the two reference methods (food frequency questionnaire and four dietary records) and useful for categorizing individuals according to their habitual intake of selected food and drink groups [37].

Only few of the reviewed studies included CB-product consumption among exclusion criteria and/or have instructed volunteers to avoid their consumption, whereas none of them have monitored the diet (Table 4).

We could not analyse the effects of different study designs (crossover/parallel), due to the low number of studies. As previously suggested [24], parallel design could lead to ineffective randomization and potential confounding, while the correct washout period in crossover design, in order to prevent a carry-over (or residual) effect, is difficult to establish in absence of bioavailability data, as in the reviewed studies (Table 4).

CB and its bioactive compounds, in vitro, inhibited enzymes and transporters involved in drug bioavailability and pharmacokinetics [38, 39]. Although only few cases have been reported [38], probably related to genetic polymorphisms in the enzymes or transporters [40], potential food-drug interactions should be monitored in individuals with SCI under treatment for NLUTD with drugs including oxybutynin and solifenacin [41]. The panel of experts of the “clinical guidelines for the diagnosis and treatment of lower urinary tract dysfunction in patients with SCI” concluded that anticholinergic (oxybutynin) and *β*3-adrenoceptor agonist (mirabegron) drugs are recommended for patients at risk of renal damage, with symptomatic UTI or urinary incontinence [10]. However, adverse anticholinergic events can occur in SCI patients, including blurred vision, dry mouth, and constipation [10]. In this context, neurogenic bowel dysfunction (NBD), comorbidity, and polypharmacy and potential nutraceutical-drug interactions should be monitored [33], in particular in aged patients with SCI [42]. With ageing, individuals with SCI have an increased risk of renal stones [42], and the effect of CB on nephrolithiasis is controversial [43, 44].

Despite the large age range of many reviewed studies (Table 2), comorbidity and drug use were not reported (Table 4), and only antimicrobials and drugs acting on the immune system were among the exclusion criteria (Table 2).

Only a study evaluated the effect of CB juice (Table 3) and did not observe differences in bacteriuria compared to water (Table 3 and Figure 2). Reid et al. [26] reported that CB juice ingestion, but not water intake, decreased the adhesion of Gram-negative (*p* = 0.054) and Gram-positive (*p* = 0.022) bacteria from the urinary sample to uroepithelial cells, despite the similar reduction of bacteriuria compared to baseline. In addition to urine culture, the authors suggested that further parameters need to be tested in patients with SCI, since urine alone is a poor indicator of bacterial colonization [26]. Accordingly, CB products do not appear to inhibit bacterial growth or sterilize the urinary tract, and among the suggested mechanisms explaining the preventive effects of CB consumption against UTI, the hypothesis concerning acidification of urine, due to the excretion of the bacteriostatic hippuric acid, has currently been disproved [45].

On the other hand, PAC, especially A-linked PAC, had an antiadhesion effect of P-fimbriated uropathogenic Escherichia coli to uroepithelial cells, whereas B-linked PAC, found in green tea, dark chocolate, grapes, and apples, did not have this antiadhesion activity [45]. It has been reported that mucosal production of interleukin-6 (IL-6) was due to an adhesion-dependent interaction of bacteria with the mucosa [46], and it has been suggested that CB may reduce UTI symptoms through anti-inflammatory mechanisms [45]. Despite this, urinary or serum cytokines have not been included among the outcomes in the reviewed studies.

### 4.3. Agreements and Disagreements with Other Reviews

The presence of mixed treatment groups (CB and CB+MH) and the low risk of incomplete outcome data (Table 1) assigned by Jepson et al. [17] to the study of Lee et al. [28], which included in the analysis also individuals who had discontinued the treatment, are in our opinion a bias in evaluating the efficacy.

As reported in the meta-analysis by Jepson et al. [17] the “gold standard” bacteriological criteria for diagnosis of UTI includes bacteriuria greater than 100,000 bacterial cfu/mL from the midstream specimen of urine, whereas for the catheter specimen of urine, a bacteriuria < 100, 000/mL is acceptable. Despite this recommendation, only one of the reviewed studies gave a different bacteriuria threshold for midstream and catheter specimens of urine (Table 2), despite the high variability in NLUTD management, due to the differences in lesion level and completeness among patients (Table 2). Cervical SCI has distinct features compared to other SCI, caused by the impairment of manual dexterity, preventing individuals from carrying out self-catheterization (complete injury above C5 and C6), and it is often accompanied by autonomic dysfunction [10]. The latter develops in patients with high-level SCI (above T6), and bladder and bowel distension are the leading causes of autonomic dysfunction [10]. Autonomic dysfunction (above T6) is among the symptoms suggestive of UTI that can be used for the diagnosis of symptomatic UTI in conjunction with significant bacteriuria and pyuria [10, 12, 13, 47]. In some of the reviewed studies, symptomatic UTI diagnosis required also autonomic dysfunction in individuals with lesions above T6. However, in some cases, symptomatic UTI was among the exclusion criteria (Table 2). This choice could be a bias for the evaluation of efficacy due to the low risk of UTI in the control group (Figure 3). Previous reviews did not consider these aspects, as well as bias included in the quality score described in Table 4.

## 5. Conclusion

### 5.1. Implication for Practice

The low quality of the reviewed studies makes it impossible to recommend or exclude the use of CB for preventing bacteriuria in individuals with SCI. On the other hand, it must be considered that fluid intake (2–2.5 L/day) is part of NBD management [33], and reductions greater than threefold of UTI incidence have been documented when NBD was reduced in individuals with SCI [47]. Therefore, in clinical practice, we suggest the evaluation of comorbidity and accurate diet and fluid intake monitoring. Previously, mobile phone applications were used for fluid and/or food intake recording and monitoring in the management of patients undergoing dialysis [48] or with diabetes [49], age-related macular degeneration [50], and cancer [51]. In addition, a risk evaluation for potential food-drug or nutraceutical-drug interactions, due to treatment for NLUTD and comorbidity, should be performed in a personalized patient-centered approach [6].

### 5.2. Implication for Research

A possible solution to overcome the observed variability among individuals (Table 2) could be the crossover design and monitoring of total polyphenols or of their metabolites identified in plasma and urine after the consumption of CB juice, including cinnamic, dihydrocinnamic, phenylacetic, benzoic, and hippuric acids; flavonols; benzaldehydes; catechols; valerolactones; and/or pyrogallols [32]. The pentacyclic triterpene ursolic acid has been suggested as the main compound in CB able to inhibit the activity of cyclooxygenase-2, and CB extracts inhibited nuclear factor *κ*B transcriptional activation in T lymphocytes and suppressed the release of IL-6, IL-1*β*, IL-8, and tumor necrosis factor-alpha (TNF-*α*) from Escherichia coli lipopolysaccharide-stimulated peripheral blood mononuclear cells [45].

Serum levels of inflammatory cytokines, including TNF-*α* and IL-6, were higher in individuals with SCI, compared to healthy controls, and were further elevated in SCI subjects with UTI [52]. In a pilot study on pregnant women, CB juice consumption reduced urinary IL-6 [53], which has been suggested for differentiating between lower UTI and pyelonephritis [54].

For future research, we suggest crossover design with appropriate washout period in order to overcome variability among subjects and to consider plasma and urinary markers of inflammation as outcomes in addition to symptomatic UTI.

## Figures and Tables

**Table 1:** Risk of bias from previous meta-analyses.

Study name	Linsenmeyer	Waites	Lee	Hess
Year	2004	2004	2007	2008
	Random sequence generation			
Jepson et al. 2012 [17]	Unclear	Unclear	Low	Unclear
Wang et al. 2012 [18]	Not reported	Unclear	Not reported	Unclear
Luis et al. 2017 [19]	Not reported	Unclear	Low	Unclear
	Allocation concealment			
Jepson et al. 2012 [17]	Unclear	Unclear	Low	Low
Wang et al. 2012 [18]	Not reported	Unclear	Not reported	Unclear
Luis et al. 2017	Not reported	Low	Low	Unclear
	Blinding of participants and personnel			
Jepson et al. 2012 [17]	Low	Low	Low	Low
Wang et al. 2012 [18]	Not reported	Low	Not reported	Low
Luis et al. 2017 [19]	Not reported	Low	Low	Low
	Blinding of outcome assessment			
Jepson et al. 2012 [17]	Low	Unclear	Low	Low
Wang et al. 2012 [18]	Not reported	Not reported	Not reported	Not reported
Luis et al. 2017 [19]	Not reported	Low	Low	Low
	Incomplete outcome data			
Jepson et al. 2012 [17]	Low	High	Low	High
Wang et al. 2012 [18]	Not reported	High	Not reported	Unclear
Luis et al. 2017 [19]	Not reported	Low	Unclear	Unclear
	Selective reporting			
Jepson et al. 2012 [17]	Low	Low	Low	Low
Wang et al. 2012 [18]	Not reported	High	Not reported	High
Luis et al. 2017 [19]	Not reported	Low	Unclear	Low
	Other bias			
Jepson et al. 2012 [17]	Unclear	Unclear	Low	Low
Wang et al. 2012 [18]	Not reported	Not reported	Not reported	Not reported
Luis et al. 2017 [19]	Not reported	Low	Low	Low

**Table 2:** Study setting, characteristics of volunteers, and missing data.

Study name	Reid	Linsenmeyer	Waites	Lee	Hess	Sappal
Year	2001	2004	2004	2007	2008	2018
Country	Canada	USA	USA	Australia	USA	USA
Setting		Urology clinic	Hosp. clinic, community residing	SCI database, community residing	Veterans Admin Hosp.	Veterans Affairs Medical Center
Inclusion criteria	SCI with UTI history	SCI with NLUTD	SCI (≥1 year) with NLUTD	SCI with NLUTD	SCI (≥1 year) with NLUTD	SCI (≥6-months) requiring catheterization
Lesion level, ASIA class	Paraplegia/tetraplegia not reported	From cervical 4 to cervical 7: 8 From thoracic 4 to thoracic 10: 7 From thoracic 11 to lumbar 1: 6	Paraplegia/tetraplegia 20/6 (treatment) 14/8 (control) complete/incomplete 23/3 (treatment) 17/5 (control)	Paraplegia/tetraplegia 62/91 (treatment) 76/76 (control) Complete/incomplete 67/86 (treatment) 81/71 (control)	ASIA A: 27 ASIA B: 10 ASIA C: 10 Para/tetra: 24/23	Cervical 5: 1; cervical 6: 3; cervical 7: 3; thoracic 1: 1; thoracic 5: 1; thoracic 7: 1; thoracic 9: 1; thoracic 11: 1 ASIA A: 9 ASIA B: 1 ASIA C: 3
Exclusion criteria	High serum creatinine, antibiotics, immunosuppressants, autonomic dysreflexia, cancer, stone, symptomatic UTI		Antimicrobial urinary acidifying agents (within 7 days) fever, chills	Antimicrobial symptomatic-UTI renal or hepatic disease Allergy	Low glomerular filtration rate, immunosuppressant malignancy	Antimicrobial symptomatic UTI (within 2 weeks) Immunocompromising (HIV, steroid, chemotherapy)
Included in UTI definition		White blood cells, symptoms (autonomic dysreflexia)	White blood cells ≥ 10/*μ*L	White blood cells ≥ 100 high power field Symptoms (autonomic dysreflexia)	White blood cells ≥ 10 high power field Symptoms (autonomic dysreflexia)	White blood cells ≥ 10 high power field
Age (years)	Mean: 42.3	Not specified	Range: 20-73	Range: 16-82	Range: 28-79	Range: 18-65
Gender	Males: 10/15	Males: 16/21	Males: 42/48	Males: 253/305	All men (47)	All men (13)
Number (analysed)	15	21	48	305	47	13
Loss to follow-up	1/16	16/37	26/74	0/305	10/57	0/13
Dropout	1/16 (6%)	16/37 (43%)	26/74 (35%)	CB: 12/78 (15%) CB+MH: 16/75 (21%) Placebo: 10/77 (13%) MH: 17/75 (23%)	10/57 (17%)	0/13

ASIA: American Spinal Injury Association; CB: cranberry; MH: methenamine hippurate; NLUTD: neurogenic lower urinary tract dysfunction; UTI: urinary tract infection.

**Table 3:** Intervention and outcomes.

Study name	Reid	Linsenmeyer	Waites	Lee	Hess	Sappal
Year	2001	2004	2004	2007	2008	2018
Study design	Longitudinal	Crossover randomized controlled	Parallel randomized controlled	Parallel (4 groups) randomized controlled	Crossover randomized controlled	Parallel randomized controlled
Intervention Cranberry	Juice 750 mL (3 × 259 mL, mealtimes) PAC content not reported	Tablets 1.2 g/d (3 × 0.4 g) PAC content not reported	Capsule 2.0 g/d PAC content not reported	Tablets 1.6 g/d CB 1.6 g/d + MH 0.2 g/d PAC content not reported	Tablet 1.0 g/d (2 × 0.5 g) PAC content not reported	Capsule PAC content 36 mg
Control	Water 750 mL (3 × 259 *mL*, mealtimes)	Placebo	Placebo identical (lactose)	Placebo and MH 0.2 g/d	Placebo identical (rice flour)	Placebo
Study duration	1 week each, 2 days washout	4 weeks each, 1 week washout	6 months	6 months	6 months	15 days
Outcomes	Bacteriuria Bacterial biofilm load	Bacteriuria, pyuria	Bacteriuria	Symptomatic UTI	Symptomatic UTI	Bacteriuria, pyuria
Bacteriuria (cut-off)	Not specified	Midstream specimen of urine: ≥10^4^/mL catheter specimen of urine: >10^5^/mL	Catheter specimen of urine: ≥10^4^/mL	≥10^5^/mL	≥10^4^/mL	≥10^5^/mL
Reported effects	Water: 7/15 CB juice: 7/15	Not significant	Treatment: 10/26 Control: 8/22	Treatment (CB and CB+MH): 67/153 Control (placebo and MH): 71/152	Treatment: 6/47 Control: 16/47	Not significant
Adverse effects	Not reported	Not reported	Not reported	Mild and infrequent	Not reported	Not reported

CB: cranberry; MH: methenamine hippurate; PAC: proanthocyanidins; UTI: urinary tract infection.

**Table 4:** Specific risk of bias assessment.

Study name	Reid	Linsenmeyer	Waites	Lee	Hess	Sappal
Year	2001	2004	2004	2007	2008	2018
Groups balanced for lesion level/urine collection	Yes 0.1	Yes 0.1	Unbalanced catheterization (treatment/control 65.4/36.3%)	No data for each of the 4 arms	Yes 0.1	Not reported
UTI diagnosis including autonomic dysreflexia	No	Yes 0.1	No	Yes 0.1	Yes 0.1	No
Comorbidity and drug use specified	No	No	No	No	No	No
Proper control	Yes 0.3	Yes 0.3	Yes 0.3	Placebo+MH	Yes 0.3	Yes 0.3
Compliance assessment	No	No	Pills' count 0.1	No	Pills' count 0.1	No
Dietary record	No	No	Only fluid intake 0.03	No	No	No
Food antioxidant intake	Only no CB/vit. C 0.025	Only no CB 0.025	Only no CB 0.025	No	No	Only no CB 0.025
Washout and/or run-in	2d-w/o only	Yes 0.05	No	No	No	No
Marker of bioavailability	No	No	No	No	No	No
Double blinding	No	Yes 0.05	Yes 0.05	Yes 0.05	Yes 0.05	Yes 0.05
No funding support	Yes 0.03 (no conflict)	Yes 0.03	Yes 0.03	Brucia Pharmaceuticals	Yes 0.03	Yes (critical versus sponsor) 0.03
No supplement donation	Yes 0.01	Kessler Pharmacy	AIM This Way	Yes 0.01	Cran-Max Swiss	Yes 0.01
Quality score (range 0–1)	0.465	0.655	0.535	0.16	0.68	0.415

CB: cranberry; MH: methenamine hippurate; UTI: urinary tract infection.

## Data Availability

The data used to support the findings of this study are available from the corresponding author upon request.

## Abbreviations

ASIA: American Spinal Injury Association
CB: Cranberry
GRADE: Grading of Recommendations Assessment, Development and Evaluation
IL: Interleukin
MH: Methenamine hippurate
NBD: Neurogenic bowel dysfunction
NLUTD: Neurogenic lower urinary tract dysfunction
NNT: Number needed to treat
PAC: Proanthocyanidins
RR: Risk ratio
SCI: Spinal cord injury
TNF: Tumor necrosis factor
UTI: Urinary tract infection.
